# Supplementary material for: Triiodothyronine Acts as a Smart Influencer on Hsp90 via a Triiodothyronine Binding Site
Source: Int J Mol Sci. 2022 Jun 28;23(13):7150. doi: 10.3390/ijms23137150 (PMC9266618; doi:10.3390/ijms23137150)
Supplement: Supplementary file 1 [file ijms-23-07150-s001.zip › ijms-1760651-supplementary.pdf]

Triiodothyronine Acts as a Smart Influencer on Hsp90 via a Triiodothyronine Binding Site

Lu Fan [1], Athanasia Warnecke [2], Julia Weder [3], Matthias Preller [3,4] and Carsten Zeilinger [1,*]

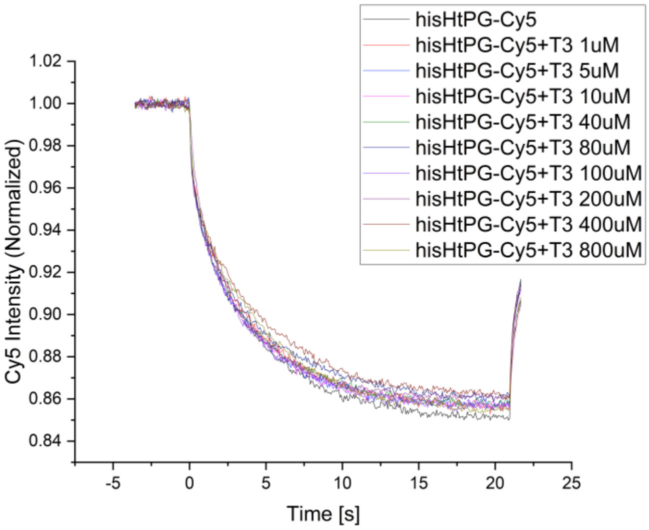

Figure S1: MST-traces of Cy5-labeled XcHtpG with increasing concentrations of T3 are displayed in the mode of thermophoresis + T-jump. Different concentrations of T3 indicate different colors of traces. Laser-induced temperature changes for F_{cold} were applied from -1 to 0 s, and for F_{hot} from 4 to 5 s.
